# Secondary Metabolites in Plants

**DOI:** 10.3390/plants14142146

**Published:** 2025-07-11

**Authors:** Javier Palazon, Miguel Angel Alcalde

**Affiliations:** Department of Biology, Healthcare and the Environment, University of Barcelona, 08028 Barcelona, Spain; miguelalcalde94@ub.edu

## 1. Introduction

**Figure 1:**
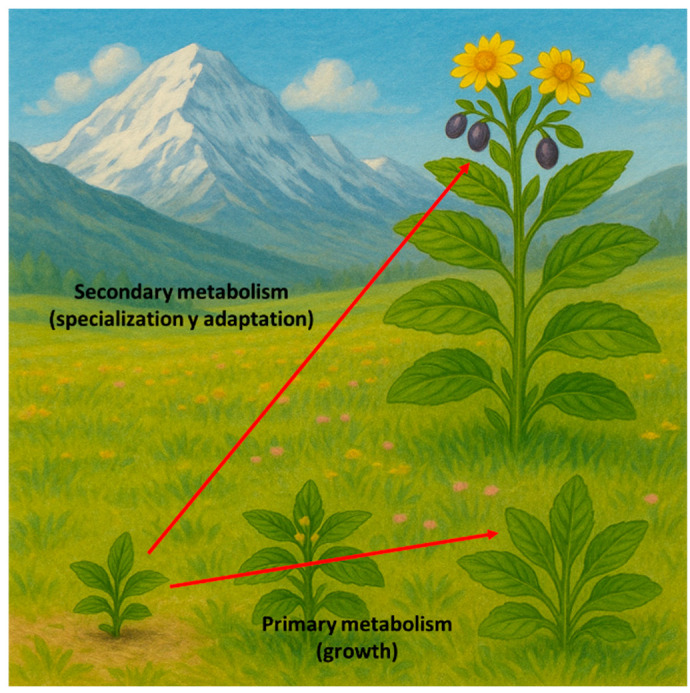
Role of primary and secondary metabolism in plant development.

Classically, plant metabolism has been divided into primary and secondary metabolism, although nowadays there is a broad interface between them that makes this classification increasingly difficult to uphold. In general, primary metabolic pathways are those present in all plant species and throughout most of a plant’s life cycle, as they are essential for survival and determine growth. In contrast, secondary metabolic pathways are not universal; the biosynthesis and accumulation of secondary metabolites (SMs) are associated with specific developmental stages, linked to the specialization of certain organs and tissues, or to particular moments in the plant’s life that are highly dependent on environmental conditions (Figure 1).

**Figure 2:**
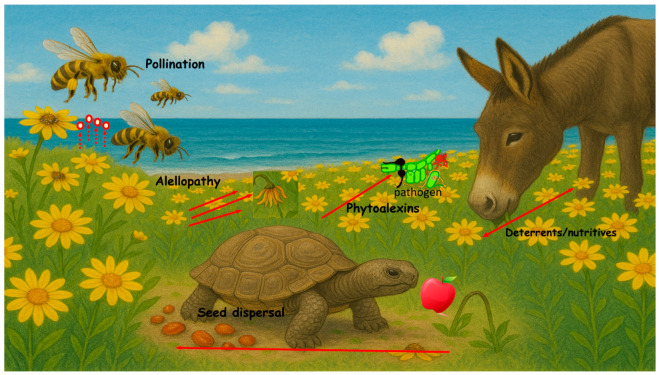
Ecological roles of the plant secondary metabolites.

Thus, it can be stated that plant secondary metabolism is a source of a plethora of chemical signals that shape plant interactions with their environment, playing key roles in processes such as pollination, seed dispersal mediated by herbivores, and defense against pathogens and other predators, among others [1] (Figure 2).

This tenuous and artificial boundary between primary and secondary metabolism implies that some vital and ubiquitous compounds in higher plants—such as phytosterols and hormones like gibberellins and abscisic acid—are produced through secondary metabolic pathways, such as the terpenoid biosynthetic route, despite their essential roles across plant taxa [2,3]. In fact, hormonal regulation, including that of abscisic acid (ABA) and salicylic acid (SA), has been shown to influence the flux of terpenoid biosynthesis under abiotic stress conditions, further blurring the line between primary and secondary metabolism [4].

**Figure 3:**
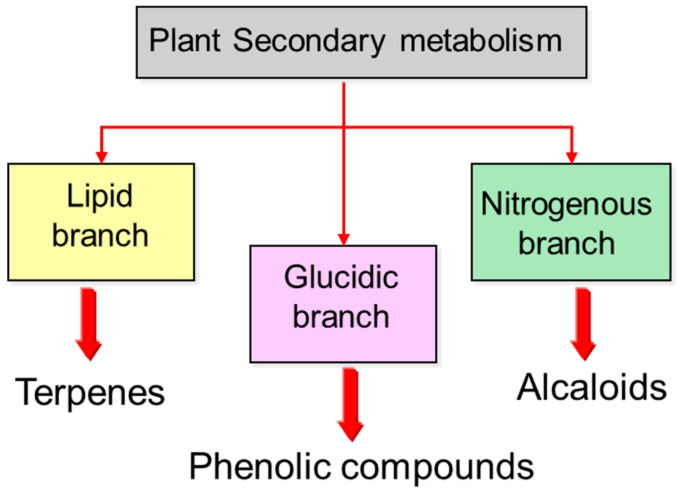
Biogenetic classification of plant secondary metabolites.

Primary metabolism supplies plants with small molecules such as simple sugars, amino acids, and fatty acids—compounds ubiquitous across the plant kingdom—which, through biochemical conversions, give rise to macromolecules like polysaccharides, proteins, nucleic acids, and lipids, which define plant structure and support growth. From these major metabolic branches—nitrogen, lipid, and carbohydrate—an immense diversity of secondary compounds emerges through specific metabolic pathways, including phenolics, terpenoids, and alkaloids, among others (Figure 3). Most of these biosynthetic pathways remain largely uncharacterized, yet their study is of vital importance, as many of the resulting compounds have strong biological activities and are widely used in the pharmaceutical, cosmetic, and food industries [5].

The growing market demand for natural plant products has threatened numerous plant species in their natural habitats and prompted the development of new strategies for sustainable production. In this context, plant biotechnology and, in particular, the development of so-called plant biofactories and the application of metabolic engineering techniques offers a more eco-sustainable alternative for producing these valuable compounds [6]. On the other hand, plant biofactories can also serve as powerful tools for elucidating secondary biosynthetic pathways, thereby expanding the range of possibilities offered by metabolic engineering to enhance the production of secondary metabolites. In this context, recent discoveries have identified novel enzymes involved in the biosynthesis of the important anticancer compound, paclitaxel (Taxol) [7].

These advances are further supported by synthetic biology efforts to reconstruct entire biosynthetic routes in heterologous systems, revealing the minimal gene sets required for paclitaxel biosynthesis and paving the way for controlled, large-scale production [8].

## 2. Special Issue Overview

This Special Issue, “Secondary Metabolites in Plants”, aims to reveal the functions and biosynthesis of SMs in plants, the biotechnological production of SMs, their biological activities, and the phytochemical characterization of plants, in search of new bioactive compounds. The Special Issue compiles fifteen contributions (eleven original articles and four reviews) that address several aspects of alkaloids, terpenes, and phenolic and volatile compounds.

### 2.1. Alkaloid Metabolism

The alkaloid accumulation in capsules of two industrial opium poppy (*Papaver somniferum*) varieties was analyzed using different capsule sizes and tissues. It revealed genotype-specific patterns of morphinane distribution. The findings highlight how structural and genetic factors influence secondary metabolite profiles. This contributes valuable knowledge to targeted breeding for enhanced alkaloid production [9].

### 2.2. Phenol Metabolism

The elicitors and genetic mutations enhance phenolic biosynthesis, with Petrova et al. [10] demonstrating yeast extract’s role in boosting caffeoylquinic acid in *Arnica montana*, while Li et al. [11] associated elevated phenolics with antioxidant activity and leaf color in a *Lagerstroemia* mutant.

Advances in enzyme evolution and regulatory networks are reshaping our understanding of phenolic biosynthesis, with Zhou et al. [12] tracing the origin of *Mentha longifolia* acyltransferases to gene duplication events, while Tan et al. [13] reveal how kinase-transcription factor interactions govern secondary metabolism in *Forsythia.*

Innovative biotechnological approaches are catalyzing the sustainable production of valuable plant metabolites, with Suprun et al. [14] identifying *Reynoutria japonica* as a superior stilbene source and developing optimized culture systems, while Sharma et al. [15] have demonstrated the potential of *Dalea purpurea* hairy root cultures to efficiently produce bioactive flavonoids with therapeutic applications.

Cutting-edge research is uncovering novel sources and applications of bioactive plant compounds, with Morante et al. [16] demonstrating the therapeutic promise of Moraceae prenylated flavonoids for drug development, while Khamsaw et al. [17] have revealed banana peel’s untapped potential as a sustainable source of health-promoting phenolics and prebiotics for functional foods.

### 2.3. Terpene Metabolism

Emerging biotechnological and ecological insights are revolutionizing terpenoid research, with Alcalde et al. [18] engineering *Centella asiatica* hairy roots for enhanced centelloside production, Cheng et al. [19] synthesizing critical knowledge on terpenoid biosynthesis pathways, and Shakeel et al. [20] advocating for saponin-based solutions in sustainable agriculture.

### 2.4. Volatile Compounds

Environmental factors and biotic interactions significantly shape plant volatile profiles, as demonstrated by Nurcholis et al. [21], who showed how shading enhances the essential oil bioactivity of Curcuma xanthorrhiza, Sun et al. [22] revealing virus-induced changes in lemon terpenes that alter insect attraction, and Charoimek et al. [23] highlighting abiotic stress effects on Rosa damascena fragrance compounds.

## 3. Concluding Remarks

The manuscripts featured in this Special Issue explore the fascinating diversity of plant secondary metabolism, offering cutting-edge insights into biosynthesis, regulation, and ecological and biotechnological applications. As Guest Editors of this Special Issue, we are grateful to the authors for their valuable contributions, which will advance our understanding of how plants produce and utilize these specialized compounds. We also extend our sincere appreciation to the reviewers for their constructive feedback and to the Editorial Office for their unwavering support in bringing this collection to fruition.

## References

[1] Li H., Chen N., Zhang H., Xu D. (2025). Multidimensional regulation of transcription factors: Decoding the comprehensive signals of plant secondary metabolism. Front. Plant Sci..

[2] Basarllo O., Lucido A., Sorribas A., Marin-Sanguino A., Vilaprinyo E., Martinez E., Eleiwa A., Alves R. (2024). Modeling the effect of daytime duration on the biosynthesis of terpenoid precursors. Front. Plant Sci..

[3] Rogowska A., Szakiel A. (2021). Enhancement of Phytosterol and Triterpenoid Production in Plant Hairy Root Cultures—Simultaneous Stimulation or Competition?. Plants.

[4] Yang M., Wang M., Zhou M., Zhang Y., Yu K., Wang T., Bu T., Tang Z., Zheng T., Chen H. (2023). ABA and SA Participate in the Regulation of Terpenoid Metabolic Flux Induced by Low-Temperature within *Conyza blinii*. Life.

[5] Bapat V.A., Kavi Kishor P.B., Jalaja N., Jain S.M., Penna S. (2023). Plant Cell Cultures: Biofactories for the Production of Bioactive Compounds. Agronomy.

[6] Devi A.M., Khedashwori D., Prem D., Das S. (2023). Metabolic engineering of plant secondary metabolites: Prospects and its technological challenges. Front. Plant Sci..

[7] Zhang Y., Wiese L., Fang H., Alseekh S., de Souza L.P., Scossa F., Fernie A. (2023). Synthetic biology identifies the minimal gene set required for paclitaxel biosynthesis in a plant chassis. Mol. Plant.

[8] Yang C., Wang Y., Su Z., Xiong L., Wang P., Lei W., Yan X., Ma D., Zhao G., Zhou Z. (2024). Biosynthesis of the highly oxygenated tetracyclic core skeleton of Taxol. Nat. Commun..

[9] Májer P., Németh É.Z. (2024). Alkaloid Accumulation and Distribution within the Capsules of Two Opium Poppy (*Papaver somniferum* L.) Varieties. Plants.

[10] Petrova M., Geneva M., Trendafilova A., Miladinova-Georgieva K., Dimitrova L., Sichanova M., Nikolova M., Ivanova V., Dimitrova M., Sozoniuk M. (2025). Antioxidant Capacity and Accumulation of Caffeoylquinic Acids in *Arnica montana* L. In Vitro Shoots After Elicitation with Yeast Extract or Salicylic Acid. Plants.

[11] Li S., Yin M., Wang P., Gao L., Lv F., Yang R., Li Y., Wang Q., Li L., Liu Y. (2024). Phenolic Compounds and Antioxidant Capacity Comparison of Wild-Type and Yellow-Leaf *gl1* Mutant of *Lagerstroemia indica*. Plants.

[12] Zhou J., Zou X., Deng Z., Duan L. (2024). Analysing a Group of Homologous BAHD Enzymes Provides Insights into the Evolutionary Transition of Rosmarinic Acid Synthases from Hydroxycinnamoyl-CoA:Shikimate/Quinate Hydroxycinnamoyl Transferases. Plants.

[13] Tan X., Chen J., Zhang J., Guo G., Zhang H., Zhao X., Lv S., Xu H., Hou D. (2023). Gene Expression and Interaction Analysis of *FsWRKY4* and *FsMAPK3* in *Forsythia suspensa*. Plants.

[14] Suprun A.R., Kiselev K.V., Aleynova O.A., Manyakhin A.Y., Ananev A.A. (2024). Analysis of Phenolic Compounds of *Reynoutria sachalinensis* and *Reynoutria japonica* Growing in the Russian Far East. Plants.

[15] Sharma A.R., Gajurel G., Abdel-Karim S., Alam M.A., Shields R.C., Medina-Bolivar F. (2025). Production of Malheuran A, a Geranylated Flavonoid with Antimicrobial and Anti-Inflammatory Activities, in Hairy Root Cultures of *Dalea purpurea*. Plants.

[16] Morante-Carriel J., Živković S., Nájera H., Sellés-Marchart S., Martínez-Márquez A., Martínez-Esteso M.J., Obrebska A., Samper-Herrero A., Bru-Martínez R. (2024). Prenylated Flavonoids of the Moraceae Family: A Comprehensive Review of Their Biological Activities. Plants.

[17] Khamsaw P., Sommano S.R., Wongkaew M., Willats W.G.T., Bakshani C.R., Sirilun S., Sunanta P. (2024). Banana Peel (*Musa* ABB cv. Nam Wa Mali-Ong) as a Source of Value-Adding Components and the Functional Properties of Its Bioactive Ingredients. Plants.

[18] Alcalde M.A., Palazon J., Bonfill M., Hidalgo-Martinez D. (2023). Enhancing Centelloside Production in *Centella asiatica* Hairy Root Lines through Metabolic Engineering of Triterpene Biosynthetic Pathway Early Genes. Plants.

[19] Cheng R., Yang S., Wang D., Qin F., Wang S., Meng S. (2025). Advances in the Biosynthesis of Plant Terpenoids: Models, Mechanisms, and Applications. Plants.

[20] Shakeel A., Noor J.J., Jan U., Gul A., Handoo Z., Ashraf N. (2025). Saponins, the Unexplored Secondary Metabolites in Plant Defense: Opportunities in Integrated Pest Management. Plants.

[21] Nurcholis W., Rahmadansah R., Astuti P., Priosoeryanto B.P., Arianti R., Kristóf E. (2024). Comparative Analysis of Volatile Compounds and Biochemical Activity of *Curcuma xanthorrhiza* Roxb. Essential Oil Extracted from Distinct Shaded Plants. Plants.

[22] Sun Y.-D., Wallis C.M., Krugner R., Yokomi R. (2025). *Citrus Yellow Vein Clearing Virus* Infection in Lemon Influences Host Preference of the Citrus Whitefly by Affecting the Host Metabolite Composition. Plants.

[23] Charoimek N., Phusuwan S., Petcharak C., Huanhong K., Prasad S.K., Junmahasathien T., Khemacheewakul J., Sommano S.R., Sunanta P. (2023). Do Abiotic Stresses Affect the Aroma of Damask Roses?. Plants.

